# Ketamine treatment for depression: a review

**DOI:** 10.1007/s44192-022-00012-3

**Published:** 2022-04-15

**Authors:** Mani Yavi, Holim Lee, Ioline D. Henter, Lawrence T. Park, Carlos A. Zarate

**Affiliations:** Experimental Therapeutics and Pathophysiology Branch, National Institute of Mental Health, National Institutes of Health [NIMH-NIH], 10 Center Dr, Room 7-5545, Bethesda, MD 20814 USA

## Abstract

This manuscript reviews the clinical evidence regarding single-dose intravenous (IV) administration of the novel glutamatergic modulator racemic (*R,S*)-ketamine (hereafter referred to as ketamine) as well as its *S*-enantiomer, intranasal esketamine, for the treatment of major depressive disorder (MDD). Initial studies found that a single subanesthetic-dose IV ketamine infusion rapidly (within one day) improved depressive symptoms in individuals with MDD and bipolar depression, with antidepressant effects lasting three to seven days. In 2019, esketamine received FDA approval as an adjunctive treatment for treatment-resistant depression (TRD) in adults. Esketamine was approved under a risk evaluation and mitigation strategy (REMS) that requires administration under medical supervision. Both ketamine and esketamine are currently viable treatment options for TRD that offer the possibility of rapid symptom improvement. The manuscript also reviews ketamine’s use in other psychiatric diagnoses—including suicidality, obsessive–compulsive disorder, post-traumatic stress disorder, substance abuse, and social anxiety disorder—and its potential adverse effects. Despite limited data, side effects for antidepressant-dose ketamine—including dissociative symptoms, hypertension, and confusion/agitation—appear to be tolerable and limited to around the time of treatment. Relatively little is known about ketamine’s longer-term effects, including increased risks of abuse and/or dependence. Attempts to prolong ketamine’s effects with combined therapy or a repeat-dose strategy are also reviewed, as are current guidelines for its clinical use. In addition to presenting a novel and valuable treatment option, studying ketamine also has the potential to transform our understanding of the mechanisms underlying mood disorders and the development of novel therapeutics.

## Background

Racemic (*R,S*)-ketamine (hereafter referred to as ketamine), first synthesized in 1962, is a rapid-acting general anesthetic; it has been on the World Health Organization’s Essential Medications List since 1985 [1]. The Food and Drug Administration (FDA) approved ketamine in 1970 as an anesthetic agent for procedures requiring no skeletal-muscle relaxation, as an induction agent preceding other general anesthetic agents, and as a supplementary agent to low-potency anesthetics. Ketamine is a dissociative anesthetic with a relatively wide safety margin and is typically used in adult and pediatric procedures as well as veterinary procedures. Structurally, it is related to phencyclidine (PCP) and primarily acts on the glutamatergic system as an N-methyl-D-aspartate (NMDA) antagonist. Compared to PCP, ketamine has a shorter duration of action and is associated with fewer behavioral and adverse effects.

Trullas and Skolnick were the first to propose that NMDA receptor pathways might be involved in behavioral changes resulting from inescapable stress [2], laying the ground work for exploring ketamine’s potential antidepressant effects. Their initial investigations demonstrated that NMDA antagonists improved depressive-like symptoms in animal models of stress, and subsequent studies confirmed that ketamine in particular had antidepressant effects in animal models [3–5]. Since then, human studies have confirmed ketamine’s rapid antidepressant effects in humans (see Sect. 3.1).

Despite the growing clinical evidence reviewed below, ketamine is FDA-approved for anesthetic purposes but not for the treatment of psychiatric conditions. Nevertheless, the paradigm shifting nature of ketamine’s effects—with antidepressant response manifesting within hours rather than weeks—furthered the discovery and research of novel compounds with mechanisms of action similar to those of ketamine. Given ketamine’s potential adverse effects—including dissociation, nausea, hypertension, and tachycardia—researchers examined ketamine’s enantiomers in an effort to reproduce its antidepressant effects while reducing adverse effects. The (*S*)-enantiomer of ketamine (esketamine) was subsequently found to be more potent than ketamine at the NMDA receptor, manifesting antidepressant properties at a lower dosage [6]. The FDA approved intranasally-administered esketamine for treatment-resistant depression (TRD) in adults in 2019 and for the treatment of major depressive disorder (MDD) with acute suicidal ideation or behavior in adults in 2020 (see Sect. 4).

## The mechanism of action underlying ketamine’s antidepressant effects

Ketamine affects multiple neurotransmitter systems, including the opioidergic, monoaminergic, glutamatergic, and muscarinic systems, as well as substance P and sigma receptors. Early work by Skolnick and colleagues implicated the glutamatergic system in depression [7], and research over the past three decades further implicated the NMDA receptor in modulating the molecular and cellular processes important for synaptogenesis and neuroplasticity [8]. A recent study demonstrated that blockade of NMDA-dependent burst activity in the lateral habenula (LHb) mediated antidepressant-like effects in animals [9], and neurochemical and functional imaging studies have also corroborated that ketamine treatment partly reverses the glutamatergic and gamma aminobutyric acid (GABA)-ergic dysfunction previously identified in individuals with depression [10]. Given ketamine’s potent NMDA receptor antagonism, its antidepressant properties were thought to be related to this activity and to a resulting increase of glutamate tone at the synapse. However, the precise mechanism of ketamine’s antidepressant activity remains elusive, and other receptor systems may also be involved.

**Fig. 1:**
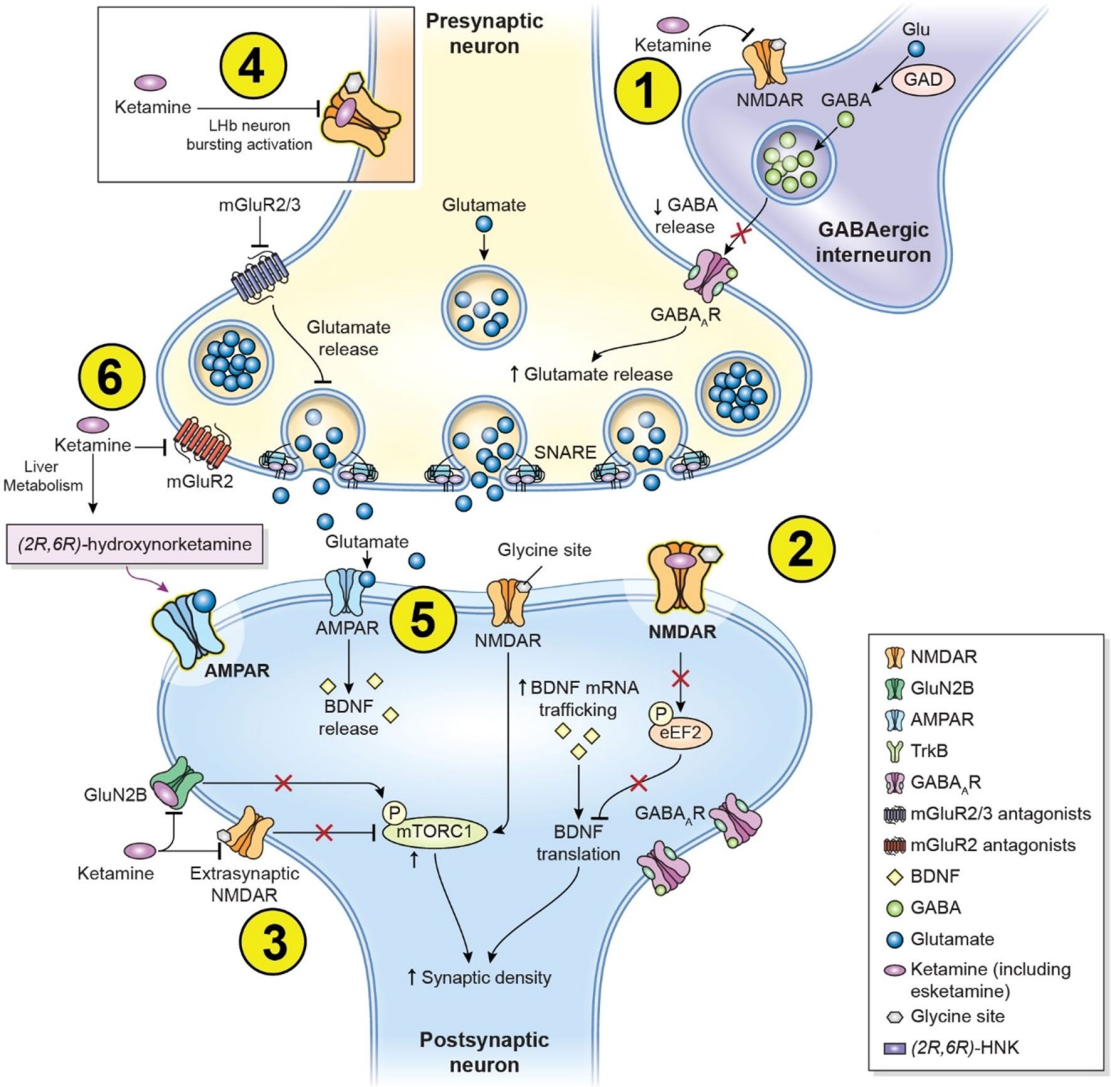
Proposed mechanisms of action of ketamine and esketamine. Ketamine’s (and esketamine’s) actions at the glutamate receptor that may mediate antidepressant effects include: (1) blocking the N-methyl-D-aspartate (NMDA) receptor at the gamma aminobutyric acid (GABA)-ergic inhibitory interneuron, leading to disinhibition of presynaptic neurons and resulting in increased glutamate release into the synapse (disinhibition hypothesis). Other downstream molecular and cellular pathways have also been investigated to better understand ketamine’s rapid acting antidepressant properties and its effects on promoting neuroplasticity. These include: (2) inhibition of synaptic and (3) extra-synaptic NMDA receptors leading to intracellular pathways promoting neuroplasticity; (4) inhibitory effects of the NMDA receptor in the lateral habenula (LHb) neurons; (5) α-amino-3-hydroxy-5-methyl-4-isoxazolepropionic acid (AMPA) receptor activation from increased synaptic glutamate release and/or ketamine metabolites (e.g., (2*R*, 6*R*)-hydroxynorketamine (HNK)); and (6) presynaptic metabotropic glutamate receptor (mGluR) (group II) antagonism leading to enhanced glutamate release and subsequent AMPA receptor activation, leading to downstream molecular and cellular pathways promoting neuroplasticity. Adapted with permission from [21].

The α-amino-3-hydroxy-5-methyl-4-isoxazolepropionic acid (AMPA) receptor [11], metabotropic glutamate receptor (mGluR) [12, 13], and opioidergic [14] signaling pathways have all been implicated in ketamine’s antidepressant properties. In addition, a growing literature has implicated the role of inflammation in MDD, and ketamine’s anti-inflammatory properties have thus gained increased consideration as another mechanism potentially underlying its antidepressant effects [15]. AMPA receptor activation in particular has been shown to modulate downstream factors, such as enhancing brain-derived neurotrophic factor (BDNF) release, which activates the tropomyosin receptor kinase B (TrkB) receptor and, subsequently, mammalian target of rapamycin complex 1 (mTORC1) [16]. Current evidence suggests that a convergence of multiple pathways may best explain ketamine’s unique therapeutic effects [17] (summarized in Fig. 1).

The variable action of ketamine’s enantiomers and respective metabolites on NMDA and AMPA receptors adds to the challenge of elucidating ketamine’s particular antidepressant effects [18], and additional downstream molecular and cellular pathways have been investigated to better understand ketamine’s rapid-acting antidepressant properties and its effects on promoting neuroplasticity [10]. In preclinical animal model studies, the ketamine metabolites (2*S*,6*S*;2*R*,6*R*)-hydroxynorketamine (HNK) were found to be essential for its rapid antidepressant effects. In addition, the antidepressant effects of the (2*R*,6*R*)-HNK enantiomer were independent of the NMDA receptor, supporting the role of AMPA receptor activity in the potentiation of excitatory synapses in mood-relevant brain regions [11]. In addition, both preclinical and clinical studies have demonstrated sex differences in the antidepressant efficacy and side effect profile of ketamine and its metabolites, further underscoring its pharmacokinetic complexity [19, 20]. Further investigations to better characterize the function of ketamine’s metabolites may lead to better isolation of its antidepressant effects with a reduced side effect profile.

This manuscript will review the clinical evidence for single-dose IV ketamine administration as well as intranasal esketamine for the treatment of MDD and TRD. A review of ketamine’s use for other psychiatric diagnoses, its potential adverse effects, and attempts to prolong its effects with combined therapy or repeated dosing will also be discussed. Finally, current treatment guidelines for ketamine’s clinical use are discussed.

## Evidence for single-infusion intravenous ketamine

### MDD

In the first double-blind, placebo-controlled human study of ketamine for the treatment of MDD, seven patients received a single intravenous (IV) infusion of ketamine (0.5 mg/kg). Compared to saline infusion, ketamine significantly improved depressive symptoms within 72 h [22]. A subsequent, adequately powered, randomized, double-blind, placebo-controlled trial of single-dose IV ketamine infusion (0.5 mg/kg) in 18 individuals with TRD found that, within two hours of infusion, ketamine significantly improved depressive symptoms compared to placebo saline infusion. The maximum effects of ketamine infusion were observed at 24 h post-infusion, with antidepressant effects lasting three to seven days [23]. Patients had undergone a psychotropic medication taper and a two-week drug-free period, and depressive symptoms were assessed using the 21-item Hamilton Depression Rating Scale (HAM-D). Later studies of single-infusion ketamine supported these findings. For instance, a meta-analysis of nine randomized, placebo-controlled studies found that ketamine had antidepressant effects that began approximately 40 min post-infusion, peaked approximately 24 h later, and lost superiority to placebo after 10–12 days [24]. Other meta-analyses have corroborated these findings [25–28].

To date, the evidence for ketamine’s antidepressant effects has been drawn from studies in adults, but a small, preliminary study was recently conducted to assess the efficacy and safety of IV ketamine infusion in adolescents with TRD [29]. In this proof-of-concept, randomized, double-blind, active placebo-controlled, crossover study, 17 adolescents aged 13–17 received either a single-dose IV ketamine infusion (0.5 mg/kg) or midazolam (an active placebo control; 0.045 mg/kg), two weeks apart. Compared to active placebo, ketamine was associated with a significantly greater reduction in depressive symptoms as measured by the Montgomery-Asberg Depression Rating Scale (MADRS). Participants experienced and tolerated transient, dissociative symptoms with ketamine. Further studies are needed to assess ketamine’s ability to treat depressive symptoms in this vulnerable population.

Finally, it should be noted that the bulk of this evidence has been collected from studies examining single, subanesthetic-dose IV ketamine administration in TRD. While other routes of ketamine administration have been explored for the treatment of MDD—including oral, rectal, intramuscular, subcutaneous, and epidural—limited data exist for these alternate routes of administration, and further study is needed to support their efficacy.

### Bipolar depression

Building on the preliminary findings in MDD, single-dose IV ketamine infusion was subsequently studied for adjunctive use with mood stabilizers in patients with treatment-resistant bipolar depression. In two randomized, placebo-controlled, crossover studies (n = 15 and n = 18), ketamine significantly improved depressive symptoms compared to placebo; effects were observed within 40 min and lasted through three days post-infusion [30, 31]. In both studies, the primary endpoint was change in MADRS scores from baseline. A subsequent, open-label study of single-dose IV ketamine infusion in 53 individuals with bipolar depression similarly demonstrated that ketamine, used in conjunction with a mood stabilizer, had rapid antidepressant effects [32].

## Evidence for intranasal esketamine

In 2019, the FDA approved intranasal esketamine, the *S*-enantiomer of ketamine, in conjunction with oral antidepressants for the treatment of TRD in adults. In 2020, it was FDA-approved to treat adults with MDD and acute suicidal ideation or behavior. Due to concerns of possible adverse effects and potential abuse, esketamine was approved through a Risk Evaluation and Mitigation Strategy (REMS). Under this agreement, intranasal esketamine can be dispensed and administered only in a REMS-certified healthcare setting under medical supervision, and patients must be monitored for at least two hours following esketamine administration.

The antidepressant efficacy of esketamine was demonstrated in two studies, both of which used a placebo solution that contained a bittering agent (denatonium benzoate) to simulate the taste of esketamine solution and maintain the blinding. In the first, randomized, placebo-controlled, double-blind study (TRANSFORM-1), 346 participants with TRD were recruited to assess the efficacy of twice-weekly intranasal esketamine (either 56 or 84 mg) plus a newly-initiated oral antidepressant [33]. Interestingly, no statistically significant difference was observed between the 84 mg intranasal esketamine plus oral antidepressant dose and the placebo nasal spray plus oral antidepressant. Unfortunately, because the predefined testing sequence limited testing of study endpoints between 56 mg intranasal esketamine plus oral antidepressant dose and placebo plus oral antidepressant, the statistically significant change in MADRS score of − 4.1 associated with the 56 mg dose could not formally be accepted for regulatory purposes.

The TRANSFORM-2 study was a four-week, randomized, placebo-controlled, double-blind Phase 3 clinical trial (n = 223) that included an antidepressant medication taper and randomization to either twice-weekly intranasal esketamine, flexibly dosed at 56 or 84 mg, or a placebo nasal spray, both administered in conjunction with a newly-initiated oral antidepressant [34]. The primary efficacy endpoint was change from baseline MADRS score. This short-term study found that, compared to intranasal placebo plus oral antidepressant, intranasal esketamine in conjunction with an oral antidepressant significantly improved depressive symptoms after four weeks by a mean difference of four points on the MADRS. The results of this Phase 3 study were instrumental in the FDA approval of esketamine for depression.

Another study (TRANSFORM-3) administered twice-weekly intranasal esketamine to 138 patients with TRD aged 65 years or older and found no significant difference in the primary endpoint of change in MADRS score after four weeks for those using intranasal esketamine (flexibly dosed at 28, 56, or 84 mg) plus a new oral antidepressant compared to placebo intranasal spray and a new oral antidepressant [35]. However, a significant change in MADRS score between the esketamine and placebo groups was noted in the subgroup analysis performed on patients from the United States and the group of participants aged 65–74 years. Further research is needed to determine whether esketamine is a good antidepressant option in elderly patients with TRD.

Another long-term, randomized, double-blind, withdrawal Phase 3 study (SUSTAIN-1) examined 297 participants who were known remitters and responders to intranasal esketamine [36]. Participants received 16 weeks of intranasal esketamine plus an oral antidepressant and were then randomized to enter a maintenance phase of either intranasal esketamine or intranasal placebo in conjunction with an oral antidepressant. In these participants who had previously achieved stable remission, 26.7% in the esketamine group relapsed during the maintenance phase compared to 45.3% in the placebo group. In the secondary analysis, the less conservative but clinically meaningful criterion of stable response (defined as a 50% or more reduction in MADRS from baseline for two weeks) was used. Of the patients who achieved stable response, 25.8% in the esketamine group and 57.6% in the placebo group relapsed, with a median time to relapse of 635 days for the esketamine group compared to 88 days for the placebo group. This study demonstrated that, in patients with TRD who achieved remission or response after esketamine treatment, continued esketamine augmentation in addition to oral antidepressants led to a clinically meaningful delay in relapse.

## Strategies to prolong ketamine’s antidepressant effects

Evidence to guide the treatment of TRD patients who had a positive initial response to ketamine remains limited. One potential strategy for prolonging the duration of ketamine’s antidepressant effects is the use of repeated ketamine infusions, which appear to be relatively well-tolerated. For instance, several studies found that ketamine’s otherwise transient antidepressant effects could be prolonged via repeated infusions [37–40]. In one study, 41 TRD participants taking concurrent standard antidepressants received a single, randomized, double-blind crossover single infusion of either ketamine or midazolam (an active placebo control) followed by six open-label ketamine infusions over two weeks [40]. The researchers found that the antidepressant effects of repeated infusions were cumulative, with the severity of depressive symptoms decreasing after each infusion. The 23 participants with a 50% decrease in MADRS score (classified as responders) received four additional weekly infusions; notably, these weekly maintenance infusions were sufficient to maintain the antidepressant effects obtained with repeated infusions. Another double-blind, randomized, placebo-controlled study of twice weekly versus thrice weekly IV ketamine administered over four weeks similarly demonstrated the effectiveness of repeated infusions in maintaining antidepressant effect; no significant difference was seen between the different dosing frequencies [39].

In contrast, another randomized, double-blind, placebo-controlled study of six repeated infusions versus a single IV ketamine infusion in patients with TRD found no significant difference in symptom remission at two weeks [41]. In another double-blind, placebo-controlled study of 26 medicated outpatients with TRD and current, chronic suicidal ideation, six ketamine infusions (0.5 mg/kg over 45 min) administered over the course of three weeks did not outperform placebo [42].

A recent study evaluated the efficacy of esketamine nasal spray (84 mg) administered twice a week versus placebo for four weeks in MDD patients with suicidal ideation in conjunction with standard care [43]. A remission rate of 47% was observed in patients who received esketamine versus 37% in the placebo group. Adverse effects, including dizziness, dissociation, nausea, dysgeusia, somnolence, headache, and paresthesia, were mostly reported on intranasal dosing days and frequently resolved on the same day, highlighting the safety profile of repeated ketamine/esketamine dosing. Taken together, the evidence suggests that despite the growing research on maintenance treatment with ketamine and esketamine, further data from adequately powered randomized trials are needed to issue standardized clinical guidance.

## Ketamine for other psychiatric indications

In recent years, ketamine has also been pursued as a potential treatment for several other psychiatric diagnoses. Despite the small number of studies and the limited sample sizes, the preliminary results from some of these studies, reviewed below, are encouraging.

### Suicidality associated with TRD

A single, subanesthetic-dose IV ketamine infusion was shown to rapidly and effectively reduce suicidal ideation in controlled trials of TRD patients [40, 44]. Another proof-of-concept, randomized, double-blind, placebo-controlled study of 18 depressed patients similarly found that a single-dose IV ketamine infusion (0.2 mg/kg) reduced suicidal ideation [45]. Notably, a systematic review and meta-analysis of 10 controlled trials of single-dose IV ketamine versus saline placebo or active midazolam control concluded that ketamine rapidly (within 24 h) reduced suicidal ideation with moderate to large effect sizes (Cohen’s d = 0.48–0.85) up to one week post-treatment [46]. In contrast, another systematic review of 25 reports from 15 independent trials (n = 572) found only moderate to low evidence that ketamine had anti-suicidal effects, but the study noted significant heterogeneity in the results and lack of data on actual suicidal behavior [47].

Intranasal esketamine was FDA-approved to treat depressive symptoms in adults with MDD with acute suicidal ideation or behavior. Two randomized, double-blind, placebo-controlled studies of adults with moderate to severe MDD with active suicidal ideation and intent compared intranasal esketamine (56 or 84 mg) versus placebo twice weekly for four weeks, in addition to comprehensive standard of care [48]. In both studies, the intranasal esketamine group demonstrated statistically greater improvement in MADRS scores than the placebo group (study 1 (n = 223), average difference in improvement = 3.8 points on the MADRS; study 2 (n = 226), average difference in improvement = 3.9 points on the MADRS). However, neither study demonstrated the superiority of intranasal esketamine over placebo for suicidal symptoms, as assessed by the Clinical Global Impression of Suicidal Severity–Revised (CGI-SS-r)). Thus, despite the improvements in depressive symptoms observed in MDD individuals with suicidality, more work is needed to determine esketamine’s effects on acute suicidality.

### Depression subtypes

Whether certain predictors of response—for instance, specific symptoms or depression subtypes—exist for ketamine remains largely unknown. Preliminary evidence suggests that anxious depression may respond better to ketamine treatment than non-anxious depression [49, 50]. In addition, both typical (melancholic) and atypical depressive symptoms appear to respond to ketamine, though typical symptoms demonstrate greater effect sizes at earlier time points (i.e., 24 h) [51]. However, additional prospective controlled studies are needed to systematically confirm these results.

### Cognitive deficits associated with MDD

Cognitive deficits in the domains of executive functioning, attention, memory, psychomotor speed, and cognitive flexibility are frequently observed in MDD and provide an important dimension to target with antidepressant treatments [52]. The number of studies investigating ketamine’s effects on cognitive function in adults with TRD or bipolar depression is limited but growing. In one double-blind, randomized, controlled clinical trial of 62 individuals with TRD, ketamine had no deleterious effect on cognitive functioning; in addition, poor processing speed at baseline was associated with improved antidepressant response to ketamine [53]. In another study of 43 individuals with TRD randomized to receive one or six ketamine infusions, researchers reported some improvements in short-term neurocognitive function, particularly in the domains of processing speed, set shifting, and spatial working memory [54]. Finally, a recent retrospective study of 68 individuals with TRD found that ketamine had pro-cognitive effects, including depression-independent improvements in working memory as measured by the Trail Making Test [55]. It should be noted, however, that the cognitive improvements that often accompany improvements in depressive symptoms have thus far limited the field’s ability to assess ketamine’s direct effects on cognition. Larger randomized controlled trials specifically designed to investigate the effects of ketamine on cognitive parameters as a primary outcome are warranted.

### Social anxiety disorder

The treatment of social anxiety disorder (SAD) with ketamine infusion was studied in a randomized, double-blind, placebo-controlled crossover study (n = 18) [56]. Participants were on stable doses of psychiatric medications, were not taking any as-needed medications for anxiety during the trial, and were not receiving cognitive behavioral therapy. IV ketamine (0.5 mg/kg) and saline placebo infusions were randomized and administered 28 days apart. Compared to placebo, ketamine significantly reduced anxiety, as assessed via the Leibowitz Social Anxiety Score (LSAS).

### Post-traumatic stress disorder

A proof-of-concept, randomized, double-blind, crossover study compared the effects of IV ketamine (0.5 mg/kg) versus the active placebo control midazolam (0.045 mg/kg) in 41 patients with chronic post-traumatic stress disorder (PTSD) [57]. The study included a two-week medication-free period and defined primary endpoint as change in PTSD symptom severity as measured by the Impact of Event Scale-Revised (IES-R). Compared to midazolam, ketamine significantly reduced PTSD symptom severity at 24 h post-infusion by a mean difference of 12.7 points on the IES-R.

Another randomized, double-blind, parallel-arm, active placebo-controlled study assessed the efficacy of repeat-dose IV ketamine infusion in 30 patients with PTSD [58]. Participants received six infusions of either IV ketamine (0.5 mg/kg) or midazolam (0.045 mg/kg) over two consecutive weeks. The primary endpoint was defined as change from baseline to two weeks in PTSD symptom severity, as measured by the Clinician-Administered PTSD Scale for DSM-5 (CAPS-5). Compared to active placebo, ketamine significantly improved PTSD symptom severity with a mean difference of 11.9 points between the ketamine and placebo groups. In ketamine responders, the median time to loss of response was 27.5 days. Although promising, more studies are needed to assess the efficacy and safety of ketamine for treating chronic PTSD.

### Obsessive compulsive disorder

A proof-of-concept, randomized, double-blind, placebo-controlled crossover study was conducted in 15 patients with obsessive–compulsive disorder (OCD) [59]. Patients were drug-free and received an IV infusion of saline or ketamine, randomized to be given one week apart. The OCD visual analog scale (OCD-VAS) and Yale-Brown Obsessive–Compulsive Scale (Y-BOCS) were used to measure OCD symptom severity. Due to carryover effects associated with ketamine, only data from the first phase of the study were analyzed. Ketamine infusion significantly improved OCD symptoms compared to placebo. Specifically, 50% of participants in the ketamine group met criteria for treatment response one week post-infusion, whereas no participants in the placebo group did.

### Substance use disorders

Preliminary data from a small number of completed studies in cocaine, opioid, and alcohol use disorders suggest that ketamine may be useful in treating substance use disorders (reviewed in [60]). Another recent, double-blind, placebo-controlled, phase 2 study of 96 individuals with severe alcohol use found that three ketamine infusions (0.8 mg/kg) per week significantly increased the number of days abstinent at three and six months compared to placebo. [61]. In a secondary analysis, the researchers also investigated the effects of combining psychotherapy and ketamine, but the effect size failed to achieve statistical significance. In this context, ketamine may prove useful in both substance use abstinence and in the management of withdrawal symptoms. However, further research is warranted to better characterize the efficacy of ketamine use for these disorders.

### Efficacy in the palliative care setting

The prevalence of MDD in patients with serious late-stage illness has been estimated to be as high as one in three. However, an estimated 75% of patients in palliative care who begin treatment with traditional antidepressants pass away just two weeks into treatment, well shy of the estimated six weeks necessary for traditional antidepressants to be effective. Consequently, the rapid antidepressant effects of ketamine have garnered much interest in the palliative care setting. Data from 11 open-label studies and one small, randomized, controlled clinical trial suggest that ketamine has robust antidepressant effects in palliative care settings, though its analgesic properties appear limited [62]. Notably, however, while ketamine’s effects on physical pain were limited at subanesthetic doses, opioid use was decreased when ketamine was used in the palliative care setting [62]. This overlap between chronic pain and depression and the underlying neurobiology has generated much interest in the opioidergic hypothesis for ketamine. More research is necessary to determine which palliative care patients would most benefit from ketamine and how to optimize their treatment.

### Eating disorders

Few studies have explored ketamine use in the treatment of eating disorders. In one open-label study of 15 patients with chronic and refractory eating disorders, ketamine was infused at 20 mg/hr for 10 h, and patients also received 20 mg of the opioid antagonist nalmefene twice daily [63]. Nine of the 15 patients responded to ketamine infusion, as characterized by return to normal eating behavior and normal weight relative to height. Responders had an average of 4.1 ± 0.8 infusions and a significant decrease in their compulsion scores.

In addition, a few case reports and case series have also explored ketamine use in the treatment of eating disorders. These include a case report of complete cessation of behavioral symptoms in bulimia nervosa at three months follow-up after three courses of IV ketamine (0.5 mg/kg over 40 min)-assisted psychotherapy [64]; a case report of remission of anorexia nervosa at six months follow-up after a series of titrated IV ketamine infusions (0.75 mg/kg to 1.2 mg/kg over 45 min) [65]; and a case series of four patients with eating disorder and TRD who received IM ketamine injections with varying degrees of improvement in eating disorder symptoms [66]. Despite these interesting preliminary results and the burgeoning interest in using ketamine to treat eating disorders, the lack of controlled clinical trials reported at this time suggests that more work is needed to better characterize ketamine’s potential for treating this patient population.

## Ketamine-assisted psychotherapy

The theory behind using ketamine in conjunction with psychotherapy is based on the notion that ketamine’s dissociative effects could allow patients greater disinhibition and cognitive flexibility that, in turn, would allow them to better engage in therapy sessions. In addition, therapies such as cognitive behavior therapy (CBT) might strengthen and maintain these ketamine-related improvements in cognitive restructuring of inaccurate beliefs and associated maladaptive information processing. To date, ketamine’s utility in enhancing psychotherapy has mainly been explored in smaller studies. One open-label study of 16 patients with TRD found that psychotherapy extended ketamine’s rapid antidepressant effects, though most responders relapsed after ceasing CBT [67]. A recent follow-up study randomized 28 patients who had achieved response after six ketamine infusions to receive either CBT or treatment as usual [67]. After 14 weeks of therapy, the investigators found a moderate, though not statistically significant, improvement in MADRS scores and a significant effect as measured by the Quick Inventory of Depressive Symptomatology, Self-Report 16-Item (QIDS-SR-16) in the CBT group. Another study of ketamine-assisted psychotherapy in 235 participants with a range of diagnoses combined data from three private practices that employed an average ketamine dose of 200–250 mg sublingually or 80–90 mg intramuscularly [68]. Patients experienced clinically significant improvements in depressive and anxiety symptoms as measured by the Beck Depression Inventory (BDI) and the Hamilton Anxiety Rating Scale (HAM-A). However, no control group was included, and the wide range of settings and methods limits these results. Larger randomized, powered studies are needed to determine whether psychotherapy-assisted ketamine treatment can sustain this agent’s antidepressant effects.

Despite the preliminary nature of the evidence described above, a small number of studies have examined the promising effects of ketamine combined with psychotherapy for treating disorders other than depression, including alcohol, heroin, cocaine, and cannabis abuse [69–72]. Ketamine was found to have benefits in treating substance addiction and dependence, increasing abstinence rates, reducing relapse rates, and decreasing cravings. Taken together, the positive findings from these preliminary studies suggest that the methodology involved in ketamine-assisted psychotherapy is presently evolving; additional studies are needed to optimize its use.

## Adverse effects of ketamine

Ketamine is classified as a Schedule III controlled substance under the Controlled Substance Act and, as noted above, approved by the FDA for anesthetic indications [73]. Common adverse effects associated with anesthetic ketamine use include emergence reactions, hemodynamic instability, and respiratory depression. These symptoms are very rarely reported at the subanesthetic doses typically administered to treat depression. At the higher doses often associated with illicit use, ketamine can induce a condition termed the “K-hole”, in which the individual may be non-communicative due to dissociative symptoms, lack of awareness of self and environment, and/or unresponsiveness to external stimuli. Symptoms are typically self-resolving, and management in these situations is supportive [74]. There is also a risk of abuse and dependence with ketamine, and repeated use may result in tolerance and dependence; individuals may experience withdrawal.

Compared to anesthetic doses (typically 1–3 mg/kg) [75, 76], the sub-anesthetic dose of ketamine used to treat TRD is considerably lower (typically 0.5 mg/kg), with a concomitant reduction in risk of adverse effects. Commonly reported side effects associated with single-dose IV ketamine infusion for TRD include psychotomimetic symptoms (e.g. dissociation, hallucinations), sympathomimetic symptoms (e.g. hypertension, tachycardia), and vestibular symptoms (e.g. nausea, vomiting, headache, dizziness) [74]. While these effects traditionally occur during infusion and are usually transient, the psychotomimetic symptoms may be distressing for some individuals who experience significant dissociative symptoms. Studies of ketamine in bipolar depression found similar self-limited psychotomimetic and dissociative symptoms that warrant close safety and tolerability monitoring [77]. It should be noted that ketamine’s dissociative effects have been investigated as potentially contributing to its antidepressant effects, particularly in the psychotherapy models discussed in Sect. 7. However, these dissociative effects have not been found to be necessary for ketamine’s antidepressant effects [78, 79].

Common adverse events associated with intranasal esketamine similarly include psychotomimetic, sympathomimetic, and vestibular symptoms. Contraindications to esketamine include aneurysmal vascular disease or arteriovenous malformation, intracerebral hemorrhage, and hypersensitivity to esketamine, ketamine, or any of their excipients [80]*.* The warning label for esketamine includes risk for sedation, dissociation, abuse, and misuse, as well as suicidal thoughts and behaviors in pediatric and young adult patients. Side effects of both ketamine and esketamine can be monitored using a questionnaire such as the Ketamine Side Effect Tool (KSET) [81]. Currently, the long-term risks and side effects of both ketamine and esketamine for TRD are not well-characterized. More evidence is needed to better understand the safety profiles of both agents.

## Guidelines and recommendations

The clinical use of ketamine is rapidly expanding, underscoring the need for standardized guidelines to direct its use. This is particularly important given that ketamine use for TRD is currently not approved by the FDA despite a significant body of evidence supporting its efficacy and safety. Interestingly, one recent study noted that, although six electroconvulsive therapy (ECT) sessions were superior to six ketamine sessions in treating TRD, both regimens were safe and effective in treating MDD [82]. Furthermore, the approval of esketamine by both the FDA and the European Medicines Agency (EMA) highlights the utility of ketamine-like agents.

The guidelines recently published by the Canadian Network for Mood and Anxiety Treatments (CANMAT) provide a comprehensive evidence-based guide for clinicians to confidently evaluate optimal treatment decisions for their patients. These guidelines gave a single infusion of IV ketamine Level 1 evidence for efficacy and recommended its use as a third-line treatment option in adults with TRD [83]. Guidelines published by *JAMA Psychiatry* [84] and, more recently, the *American Journal of Psychiatry (AJP)* [85] also underscore the strong evidence for ketamine’s antidepressant efficacy and provide detailed recommendations for its clinical use with necessary precautions to ensure patient safety. The relative efficacy of IV racemic ketamine versus intranasal esketamine has not yet been established in adequately designed head-to-head trials.

The evidence for multiple infusions—either in acute series or as ongoing maintenance treatment—is more limited and was characterized accordingly as Level 3 in the CANMAT guidelines. The limited data reflect in part that ketamine is used off-label, and that there is therefore less incentive and funding available to support conducting large, controlled continuation and maintenance studies with this agent. Current CANMAT guidelines suggest that the need for repeated and maintenance ketamine infusions should be carefully assessed on a case-by-case basis to consider potential risks and benefits. A recent review of the available evidence supporting current strategies for extending and maintaining antidepressant response to ketamine suggested several options, including repeated continued treatments with either IV ketamine, intranasal esketamine, or oral ketamine; gradually decreasing the frequency of maintenance ketamine dosing while switching treatment to traditional antidepressants, mood stabilizers, or ECT; and integrating psychotherapy and psychosocial interventions into the treatment plan [86]. Although the evidence is limited to expert recommendations, clinical experience, and preliminary research evidence, this discussion can help clinicians currently pondering effective treatment strategies for maintaining antidepressant response.

Finally, it should be noted that the CANMAT guidelines determined the evidence for non-IV formulations of racemic ketamine to be limited to Level 3 or 4. The *AJP* guidelines also compare the routes of administration for ketamine versus esketamine, their clinical dose ranges, and their bioavailibities. Given the limited evidence regarding the efficacy of oral and other routes of administration and the risk for misuse and diversion, the use of racemic ketamine should be limited to specialists with ketamine-prescribing expertise who are affiliated with tertiary or specialized centers. At present, esketamine and ketamine should be administered in clinical settings with sufficient means of monitoring patients and providing immediate care if necessary.

## Future directions

A growing body of scientific research supports the rapid antidepressant and anti-suicidal effects of ketamine in treating TRD and bipolar depression. In particular, clinical use of ketamine is rapidly expanding, despite the lack of sufficient data and few standardized guidelines to direct its use, particularly for maintenance treatment and for other formulations such as sublingual, oral, and intramuscular. Larger studies of repeat-dose administration and long-term treatment data are both needed to inform evidence-based practice guidelines. Further evidence is needed to better understand ketamine’s safety profile over longer periods of time, and its use should continue to be reserved for patients who have failed to respond to multiple existing treatment options.

Despite these challenges, studying the mechanistic processes and biomarkers that underlie ketamine’s unique properties has fundamentally changed our understanding of the pathophysiology of mood disorders [87]. Efforts to elucidate ketamine’s mechanism of action have focused attention on several areas, including: glutamatergic receptors (e.g., AMPA, mGluR); mediation via opioidergic mechanisms; the interplay between the glutamatergic and GABA-ergic systems; and downstream effects on signal transduction cascades such as mTOR, cellular proliferation, and neuroplasticity cascades [88]. This paradigm shift has expanded our vision of depressive disorders as disorders of neuroplasticity rather than merely dysfunctions in monoamine neurotransmitter systems. Furthermore, in the search to identify or develop agents whose mechanism of action mirrors ketamine’s but are not associated with its side effects or risk of abuse has directly led to the development of promising novel therapeutics. In addition to the FDA approval of esketamine, ketamine-related agents under investigation include arketamine, the (*R*)-enantiomer of ketamine, and (2*R*,6*R*)-hydroxynorketamine (HNK), a ketamine metabolite. Both are being studied in Phase 1 human trials. Notably, animal studies found that arketamine has more potent, longer-lasting antidepressant effects than either ketamine or esketamine with fewer behavioral side effects and lower abuse potential [11].

## Conclusion/summary

As reviewed above, strong evidence supports the rapid, although temporary, antidepressant and anti-suicidal effects of a single IV ketamine infusion for TRD and bipolar depression. In addition, investigations into ketamine’s effectiveness for other disorders or subtypes, alternate formulations, and its use in conjunction with therapy all require additional study and must be reproduced consistently before being added to standardized treatment guidelines. Nevertheless, the paradigm-shifting nature of the antidepressant response associated with IV ketamine and intranasal esketamine, their growing use in the community, and the evidence-based treatment recommendations for their use in mood disorders all underscore the importance of this novel addition to the treatment armamentarium for TRD.

## Data Availability

Not applicable.
